# Potentially Modifiable Dementia Risk Factors in Canada: An Analysis of Canadian Longitudinal Study on Aging with a Multi-Country Comparison

**DOI:** 10.14283/jpad.2024.105

**Published:** 2024-06-07

**Authors:** S. Son, M. Speechley, G. Y. Zou, M. Kivipelto, F. Mangialasche, H. H. Feldman, H. Chertkow, S. Belleville, H. Nygaard, V. Hachinski, F. Pieruccini-Faria, Manuel Montero-Odasso

**Affiliations:** 1 Department of Epidemiology and Biostatistics, Schulich School of Medicine & Dentistry, University of Western Ontario, 1465 Richmond Street, London, ON Canada N6G 2M1; 2 Gait and Brain Lab, Parkwood Institute, Lawson Health Research Institute, 550 Wellington Road, London, ON Canada N6C 0A7; 3 Robarts Research Institute, University of Western Ontario, 1151 Richmond St, London, ON Canada N6A 3K7; 4 Division of Clinical Geriatrics, Center for Alzheimer Research, Department of Neurobiology, Care Sciences and Society, Karolinska Institutet, Karolinska Vägen 37 A, 171 64 Solna, Sweden; 5 Neuroepidemiology and Ageing Research Unit, School of Public Health, Imperial College London, St Dunstan’s Road, London, UK W6 8RP; 6 Theme Inflammation and Aging, Medical Unit Aging, Karolinska University Hospital, Hälsovägen 7, Huddinge, 141 86 Stockholm, Sweden; 7 Research and Development Unit, Stockholms Sjukhem, Mariebergsgatan 22, 112 19 Stockholm, Sweden; 8 Alzheimer’s Disease Cooperative Study, University of California San Diego, 9500 Gilman Drive, La Jolla, CA 92037-0948 USA; 9 Department of Neurosciences, University of California San Diego, 9375 Gilman Dr, La Jolla, CA 92161 USA; 10 Kimel Centre for Brain Health and Wellness and Anne & Allan Bank Centre for Clinical Research Trials, Baycrest Hospital, 3560 Bathurst St, Toronto, ON Canada M6A 2E1; 11 Canadian Consortium on Neurodegeneration in Aging (CCNA), Administration, 3560 Bathurst St, Toronto, ON Canada M6A 2E1; 12 Research Center, Institut Universitaire de Gériatrie de Montréal, 4565 Queen Mary Rd, Montréal, QB Canada H3W 1W5; 13 Department of Psychology, Université de Montréal, 90 avenue Vincent d’Indy, Montréal, QB Canada H2V 2S9; 14 Division of Neurology and Djavad Mowafaghian Centre for Brain Health, University of British Columbia, 2215 Wesbrook Mall, Vancouver, BC Canada V6T 1Z3; 15 Department of Clinical Neurological Sciences, Schulich School of Medicine and Dentistry, University of Western Ontario, 1151 Richmond St, London, ON Canada N6A 3K7; 16 Department of Medicine, Division of Geriatric, Schulich School of Medicine and Dentistry, University of Western Ontario, 550 Wellington Rd, London, ON Canada N7C 0A7; 17 Departments of Medicine (Geriatrics) and Epidemiology and Biostatistics, Schulich School of Medicine & Dentistry, University of Western Ontario, 500 Wellington Road, London, ON Canada N6C 0A7

**Keywords:** Dementia, prevention, risk reduction, lifestyle, CLSA

## Abstract

**Background:**

It has been suggested that up to 40% of dementia cases worldwide are associated with modifiable risk factors; however, these estimates are not known in Canada. Furthermore, sleep disturbances, an emerging factor, has not been incorporated into the life-course model of dementia prevention.

**Objective:**

To estimate the population impact of 12 modifiable risk factors in Canadian adults including sleep disturbances, by sex and age groups, and to compare with other countries.

**Design:**

Cross-sectional analysis of Canadian Longitudinal Study on Aging baseline data.

**Setting:**

Community.

**Participants:**

30,097 adults aged 45 years and older.

**Measuremments:**

Prevalence and Population Attributable

**Fractions (PAFs) associated with less education, hearing loss, traumatic brain injury, hypertension, excessive alcohol, obesity, smoking, depression, social isolation, physical inactivity, diabetes, and sleep disturbances:**

**Results:**

The risk factors with the largest PAF were later life physical inactivity (10.2%; 95% CI, 6.8% to 13%), midlife hearing loss (6.5%; 3.7% to 9.3%), midlife obesity (6.4%; 4.1% to 7.7%), and midlife hypertension (6.2%; 2.7% to 9.3%). The PAF of later life sleep disturbances was 3.0% (95% CI, 1.8% to 3.8%). The 12 risk factors accounted for 51.9% (32.2% to 68.0%) of dementia among men and 52.4% (32.5% to 68.7%) among women. Overall, the combined PAF of all risk factors was 49.2% (31.1% to 64.9%), and it increased with age.

**Conclusion:**

Nearly up to 50% of dementia cases in Canada are attributable to 12 modifiable risk factors across the lifespan. Canadian risk reduction strategies should prioritize targeting physical inactivity, hearing loss, obesity, and hypertension.

**Electronic Supplementary Material:**

Supplementary material is available in the online version of this article at 10.14283/jpad.2024.105.

## Introduction

**W**ith rapid global population aging, the number of individuals living with dementia worldwide is expected to triple, from 57 million to 152 million, by 2050 (1). In Canada, dementia prevalence is projected to increase by 187% to 1,712,400 by 2050 (2). Dementia is a multifactorial syndrome that results from multiple pathologies, including those that cause neurodegeneration as well as vascular, metabolic, and inflammatory processes that are associated with potentially modifiable risk factors (3, 4). Lifestyle interventions offer a promising non-pharmacological approach to reducing dementia burden by tempering modifiable risk factors. Risk reduction can potentially be achieved through individual and public health approaches, which could complement emerging disease-modifying treatments directed at the pathological processes (4).

The 2020 Lancet Commission Report on Dementia Prevention, Intervention, and Care (5) indicated that up to 40% of dementia cases worldwide are attributable to 12 modifiable factors comprising health behaviours, illnesses, and environmental exposures across the lifespan, known as the life course model of dementia prevention. This conclusion was reached by estimating the weighted population attributable fraction (PAF), which quantifies the contribution of a given risk factor by combining both prevalence and the association between risk factor and disease, such as risk ratio, while adjusting for intercorrelation among risk factors.

Following the Lancet series (5, 6), the population impact of dementia risk factors has been estimated in other countries with differences in risk factor profiles (7–12). Assuming homogeneity of risk ratios among countries, the differences in PAF across countries are mainly driven by differences in risk factor prevalence. For instance, less education is a greater contributing factor to dementia than social isolation in low-income countries, as they tend to have stronger social ties and social support but have limited access to education compared to high-income countries (9). Despite being one of the high-income countries with a universal health care system, Canada urgently needs strategies for dementia risk reduction, as it is reaching the super-aged country status, with ≥ 20% of the population composed of older adults (13). However, no studies have estimated the population impact of the life-course model of modifiable risk factors for dementia in Canada.

Besides the 12 risk factors included in the life course model of dementia prevention, the Lancet Commission Reports also identified sleep and diet as emerging risk factors (5, 6). Given the number of intervention trials delivering sleep and diet interventions to improve cognition, it is timely to quantify their population impact (14–16). Estimating the population impact of diet is challenging due to the difficulty in measuring the diet pattern. For sleep, a recent study estimated that 5.7% of dementia cases are attributable to unhealthy sleep duration, using UK Biobank data (17). However, the population impact of sleep disturbances encompassing other conditions has not been estimated nor included in the life course model of dementia prevention.

Although air pollution is included in the life-course model (5), focusing on lifestyle risk factors, rather than including an environmental risk factor, would be more beneficial in guiding the dementia risk reduction program.

Therefore, to provide evidence to help inform future lifestyle interventions in Canada, we aimed: i) to estimate the prevalence and potential population impact of modifiable risk factors, including sleep disturbances, using the largest Canadian population cohort study, and ii) to compare the contribution of modifiable risk factors with other countries. To further provide evidence for tailoring prevention strategies, we aimed to estimate these measures stratified by age groups and sex.

## Methods

This was a cross-sectional analysis of baseline data from the Canadian Longitudinal Study on Aging (CLSA) Comprehensive cohort (18). We followed STROBE guidelines for a cross-sectional study.

### Data source

CLSA is a prospective cohort study following 51,388 Canadians aged 45–85 years at recruitment for 20 years (18). Participants were recruited to either Tracking or Comprehensive cohorts, which differed in sampling methods and data availability, as described elsewhere (18) and in Figure S1 in Appendix. The comprehensive cohort was used as it included participants who had undergone detailed and comprehensive face-to-face assessments that included a neurocognitive battery, sensory-vision, audiometry, proprioception testing, as well as physical and mobility assessments. Individuals with a diagnosis of cognitive impairment and or dementia, full-time members of the armed forces, residents of First Nations reserves, territories, or long-term care institutions (only those that provide 24-hours of nurse care), and those who could not respond in English or French at recruitment were excluded (18). Baseline data were collected from 2012 to 2015 (18).

### Risk factors

A total of 12 risk factors were identified using operational definitions from the Lancet report (5) and 2 initial PAF estimation studies for dementia (19, 20). Less education was defined as having less than secondary school graduation. Hearing loss was derived from an average hearing level of >25 dB at 500, 1000, 2000 and 4000 Hz in the better ear (21). Traumatic brain injury was defined as having at least one head injury caused by a vehicular crash, fall, or sports-related activities that resulted in losing consciousness. An average systolic blood pressure ≥140 mmHg across six seated measurements, excluding the first reading, or self-reported diagnosis was used to define hypertension. The number of drinks per week was converted to the unit of alcohol using the alcohol unit conversion formula by UK National Health Service, (Strength (ABV%)×Volume (mL))/1,000 (22). Based on the converted unit of alcohol, >21 units was used to indicate excessive alcohol use. BMI of ≥30 kg/m^2^ was used to categorize obesity. Daily or occasional cigarette smoking in the past 30 days was used to represent current cigarette smoking. Depression was categorized based on the self-reported diagnosis of clinical depression. Social isolation was defined as having less than one social contact within a month with family, friends, or neighbours. The level of physical activity was measured using the Physical Activity Scale for the Elderly (PASE) questionnaire. The total hours of physical activity per week were computed using the lowest point of each frequency and duration category, with the exception that the midpoint was used for the lowest frequency category. Based on the estimated minutes of moderate-to-vigorous physical activity, physical inactivity was defined as <150 minutes per week. A self-report diagnosis of type 2 diabetes by a physician was used for diabetes. We characterized sleep disturbances based on the definition used in the systematic review (23) from which we used their reported relative risk for PAF calculation. Sleep disturbances were broadly defined to encompass poor sleep quality, daytime sleepiness, insomnia symptoms, obstructive sleep apnea symptoms, and restless leg syndrome symptoms, based on self-reported questionnaires on sleep quality and behaviours. More details on sleep disturbances classification are provided in Table S2 Appendix.

### Statistical Analysis

Demographic characteristics were summarized using mean and standard deviation or frequency and percentage, where appropriate. To build the life-course model, the prevalence of midlife and later life risk factors was calculated for the age group 45–64 and 65–85, respectively. Early life risk factors were calculated for all age groups. The prevalence estimates were weighted with inflation weights to account for differences in selection probabilities (24).

As described elsewhere (9), PAF of each risk factor was calculated using Levin’s formula and risk factor overlap was adjusted by applying Norton’s formula (20), which involves weighting by communalities from principal component analysis (see Table S3 in the Appendix for a detailed description). Risk ratio was taken from the Lancet report (5) for all risk factors except sleep disturbances, which was derived from a recent meta-analysis of 18 longitudinal studies with an average of 9.5 years of follow up assessing the association between sleep disturbances and dementia (23).

To compare our results with global estimates (5) and other countries (7–11), we obtained the prevalence and PAF from other studies that utilized the Lancet (5) approach. The life-course model obtained from CLSA was qualitatively compared to the global estimates (5), USA (7), New Zealand (8), India (9), Latin America (9), China (9), Australia (10), Brazil (11), and Denmark (12). A total of nine risk factors were available across all the studied countries - less education, hearing loss, hypertension, obesity, smoking, depression, social isolation, physical inactivity, and diabetes - and were measured using similar definitions across studies.

To further explore differences in risk factor distribution by age groups and sex, analyses were stratified by four age groups (45–54, 55–64, 65–74, and 75–85) and sex, and chi-square tests were conducted. A sensitivity analysis compared changes in prevalence and PAF with different risk factor definitions. Depression was re-operationalized as a 10-item Center for Epidemiologic Studies Depression Scale score of ≥10 to reflect depressive symptom. Excessive alcohol use was re-defined using Canadian guidance, which is drinking ≥7 standard drinks per week (25). A five-point Steptoe social isolation index was derived to incorporate cohabitation, social contact and participation into social isolation with a cut-off of ≥3. All the analyses were conducted with R-packages survey and psy in R Version 4.2.0 (26).

## Results

A total of 30,097 participants were included (Table 1). The mean age was 59.7 years (SD 10.3) and 52% were women. The majority were white (94%), living in urban areas (90%), and 74% were married.

**Table 1:** Participant characteristics (Weighted N = 3,812,085)

	**Overall (n=30,097)**
Age, years	59.7 (10.3)
Age groups	
45 – 54	7,595 (39%)
55 – 64	9,856 (31%)
65 – 74	7,362 (18%)
75+	5,284 (12%)
Sex	
Women	15,320 (52%)
Men	14,777 (48%)
Ethnicity	
Non-white	1,326 (6.2%)
White	28,771 (94%)
Education	
< secondary school education	1,643 (17%)
Secondary school graduation	2,839 (12%)
Some post-secondary education	2,238 (9.1%)
Post-secondary degree/diploma	23,327 (62%)
Marital	
Single (never married/lived with a partner)	2,654 (8.7%)
Married/Common-law relationship	20,651 (74%)
Widowed/Divorced/Separated	6,784 (17%)
Income	
<$20,000	1,566 (6.9%)
$20,000 – $50,000	6,360 (23%)
$50,000 – $100,000	9,907 (33%)
$100,000 – $150,000	5,524 (20%)
>$150,000	4,799 (17%)
Province	
Alberta	2,957 (10%)
British Columbia	6,254 (28%)
Manitoba	3,113 (7.2%)
Newfoundland and Labrador	2,214 (2.1%)
Nova Scotia	3,078 (3.8%)
Ontario	6,418 (17%)
Quebec	6,063 (32%)
Region	
Pacific	6,254 (28%)
Prairie	6,070 (18%)
Central	12,481 (48%)
Atlantic	5,292 (5.8%)
Rurality	
Rural	2,424 (5.2%)
Urban	26,461 (90%)
Peri-urban	1,212 (4.3%)

Data shown are mean (SD) or n (%)

### Prevalence

The most prevalent risk factor was later life physical inactivity (83%) (Table 2), followed by later life sleep disturbances (40%), midlife obesity (31%), midlife hypertension (30%), and midlife hearing loss (21%). The least common risk factors were midlife smoking (6.2%) and social isolation (1.6%) in later life. The prevalence of physical inactivity was much higher than in other countries, which ranged from 15.3% in India to 82% in Australia, as shown in Table 3. Smoking and social isolation were noticeably less prevalent than in other countries.

**Table 2:** Prevalence and population attributable fraction for 12 potentially modifiable risk factors for dementia in Canada

	**Risk factors**	**RR**	**Communality (%)**	**Prevalence (%)**	**Unweighted PAF (%)**	**Weighted PAF (%)**
Early life	Less education	1.6 (1.3, 2.0)	56.3	14.0	7.8	3.2 (1.9, 4.3)
Midlife	Hearing loss	1.9 (1.4, 2.7)	52.3	21.0	15.9	6.5 (3.7, 9.3)
	Traumatic brain injury	1.8 (1.5, 2.2)	18.3	15.0	10.7	4.4 (3.3, 5.4)
	Hypertension	1.6 (1.2, 2.2)	55.4	30.0	15.3	6.2 (2.7, 9.3)
	Excessive alcohol	1.2 (1.1, 1.3)	43.5	11.0	2.2	0.9 (0.5, 1.1)
	Obesity	1.6 (1.3, 1.9)	57.1	31.0	15.7	6.4 (4.1, 7.7)
Later life	Smoking	1.6 (1.2, 2.2)	51.7	6.2	3.6	1.5 (0.6, 2.4)
	Depression	1.9 (1.6, 2.3)	22.3	12.0	9.8	4.0 (3.2. 4.8)
	Social isolation	1.6 (1.3, 1.9)	29.8	1.6	1.0	0.4 (0.2, 0.5)
	Physical inactivity	1.4 (1.2, 1.7)	54.1	83.0	24.9	10.2 (6.8, 13.0)
	Type 2 diabetes	1.5 (1.3, 1.8)	40.0	13.0	6.1	2.5 (2.4, 3.3)
	Sleep disturbances	1.2 (1.1, 1.3)	24.6	40.0	7.4	3.0 (1.8, 3.8)
	Combined PAF					49.2 (31.1, 64.9)

Abbreviations: PAF, population attributable fraction; RR, risk ratio

### PAF

**Figure 1:**
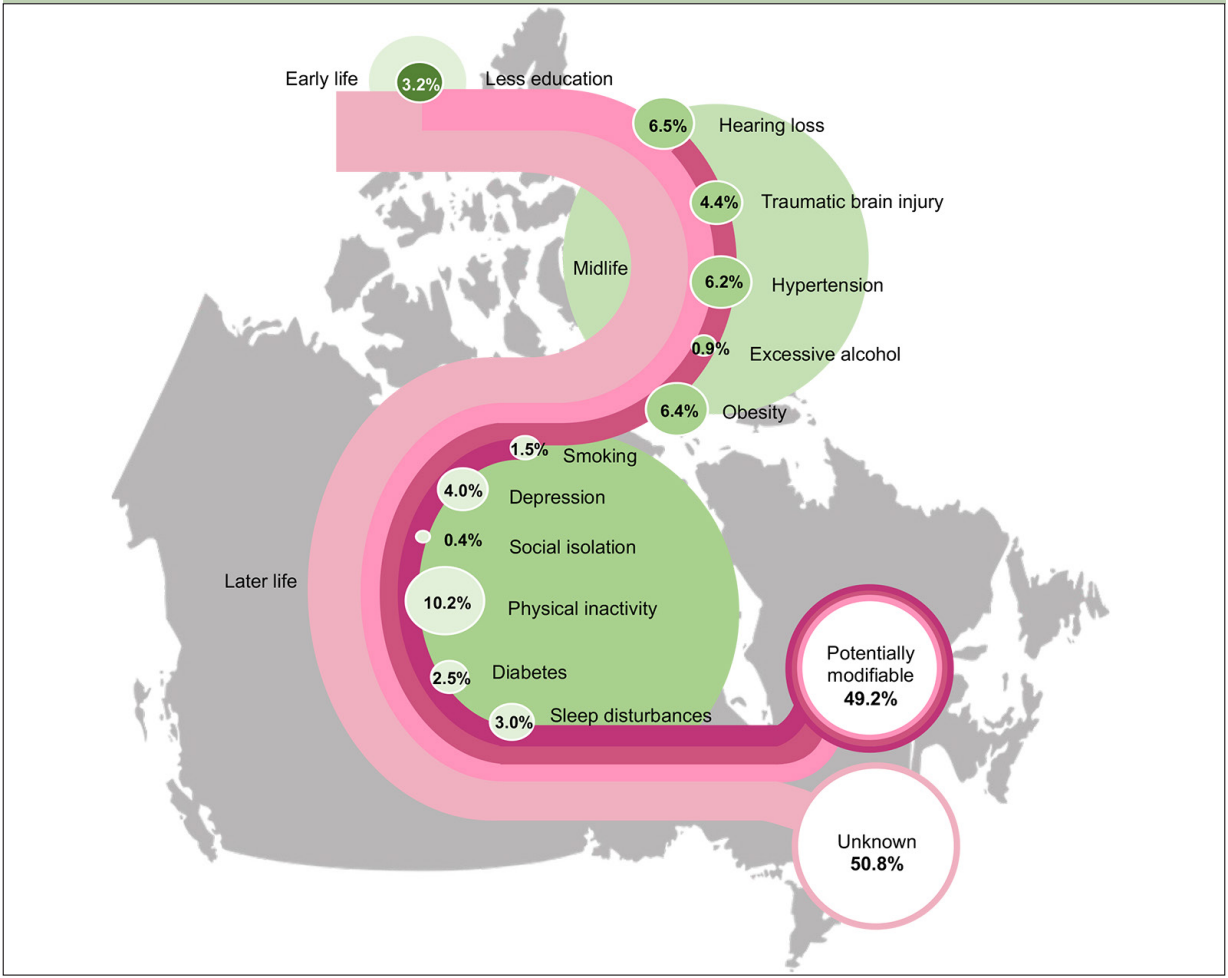
Weighted population attributable fraction for 12 potentially modifiable risk factors for dementia in Canada

The weighted individual and combined PAF of all risk factors in Canada are presented in Table 2 and Figure 1. Almost 50% of dementia cases in Canada were attributed to 12 risk factors (PAF 49.2%, 95% CI 31.1% to 64.9%) – nearly 12 points more than the worldwide estimate of 37.4% (Table 3 and Figure S2 in Appendix). The PAF of the nine risk factors available in other studied countries ranged between 33.2% and 55.9%. When using these nine risk factors, the combined PAF in Canada (41%) was higher than in USA (36%), Denmark (33.2%) and Australia (35.5%) but lower than in Latin America (55.9%). The combined PAF of the nine risk factors in Canada was similar to New Zealand, India, China, and Brazil, with a range from 39.5% and 42.1% (Table 3).

**Table 3:** Prevalence and weighted population attributable fraction of 12 potentially modifiable dementia risk factors over the life course in Canada compared to the estimates reported in other countries

		**Canada**		**Global (5)**		**US (7)**		**New Zealand (8)**		**Australia (10)**		**Denmark (12)**		**India (9)**		**Latin America (9)**		**China (9)**		**Brazil (11)**	
	**Risk factors**	**Prev (%)**	**wPAF (%)**	**Prev (%)**	**wPAF (%)**	**Prev (%)**	**wPAF (%)**	**Prev (%)**	**wPAF (%)**	**Prev (%)**	**wPAF (%)**	**Prev (%)**	**wPAF (%)**	**Prev (%)**	**wPAF (%)**	**Prev (%)**	**wPAF (%)**	**Prev (%)**	**wPAF (%)**	**Prev (%)**	**wPAF (%)**
Early life	Less education†	14.0	3.2	40.0	7.1	10.7	2.0	31.0	4.6	10.0	1.9	9.5	1.9	92.2	13.6	68.8	10.9	75.9	10.8	46.6	7.7
Mid life	Hearing loss†	21.0	6.5	31.7	8.2	10.8	3.0	39.9	7.8	26.6	7.0	23.1	6.4	22.3	6.4	28.8	7.7	14.3	3.9	26.5	6.8
	Traumatic brain injury	15.0	4.4	12.1	3.4	17.1	4.1	18.2	3.7	n/a	n/a	4.7	1.4	n/a	n/a	n/a	n/a	n/a	n/a	12.1	3.1
	Hypertension†	30.0	6.2	8.9	1.9	42.2	6.8	33.5	4.9	17.2	3.3	22.5	4.3	19.3	4.0	56.6	9.3	38.1	6.4	46.4	7.6
	Excessive alcohol	11.0	0.9	11.8	0.8	3.6	0.2	9.8	0.6	8.2	0.5	9.4	0.6	n/a	n/a	n/a	n/a	n/a	n/a	4.3	0.3
	Obesity†	31.0	6.4	3.4	0.7	44.0	7.1	38.4	5.5	38.8	6.6	22.7	4.3	13.7	2.9	44.8	7.9	32.0	5.6	31.4	5.6
Later life	Smoking†	6.2	1.5	27.4	5.2	8.5	1.7	13.5	2.2	6.7	1.4	12.2	2.4	39.9	6.4	30.0	5.7	23.0	4.2	10.6	2.1
	Depression†	12.0	4.0	13.2	3.9	7.4	2.1	19.1	4.3	11.6	3.3	8.0	2.4	5.2	1.7	23.9	6.6	1.5	0.5	15.8	4.4
	Social isolation†	1.6	0.4	11.0	3.5	11.9	2.3	37.3	5.4	5.2	1.0	8.3	1.6	10.4	2.3	0.5	0.1	3.4	0.7	1.6	0.3
	Physical inactivity†	83.0	10.2	17.7	1.6	62.8	6.8	53.6	5.2	82.0	8.3	67.6	7.5	15.3	2.2	34.2	4.5	50.7	5.8	36.7	4.5
	Diabetes†	13.0	2.5	6.4	1.1	28.6	4.2	11.6	1.6	16.8	2.7	13.2	2.4	9.3	1.7	18.5	3.2	9.4	1.6	19.7	3.1
	Sleep disturbances	40.0	3.0	n/a	n/a	n/a	n/a	31.0	4.6	n/a	n/a	n/a	n/a	n/a	n/a	n/a	n/a	n/a	n/a	n/a	n/a
Combined PAF of 9 risk factors†			40.9		33.2		36.0		41.5		35.5		33.2		41.2		55.9		39.5		42.1
Combined PAF of all risk factors			49.2		37.4		40.3		45.8		36.0		35.2		41.2		55.9		39.5		45.5

n/a Not available; Prev, Prevalence; wPAF, Weighted population attributable fraction; * Weighted PAF was calculated based on the data provided in Lee et al. (2022) (7); † Risk factors available across all studies

Of the 12 risk factors, later life physical inactivity had the largest weighted PAF in Canada, contributing 10.2% (95% CI 6.8% to 13.0%). The weighted PAF of midlife hearing loss, obesity, and hypertension in Canada were 6.5%, 6.4%, and 6.2% respectively (95% CI 3.7% to 9.2% for hearing loss, 95% CI 4.1% to 7.7% for obesity, and 95% CI 2.7% to 9.3% for hypertension). Later life smoking, midlife excessive alcohol use, and later life social isolation were associated with less than 2% of PAF in Canada. Compared to other countries, Canadian weighted PAFs were smaller for later life smoking and social isolation, while larger for later life physical inactivity, midlife traumatic brain injury and excessive alcohol use (Table 3 and Figure S2 in Appendix). The weighted PAFs for early life less education, midlife hypertension, and later life depression in Canada were larger than in other high-income countries, whereas the weighted PAF for early life less education and midlife hypertension was smaller than low- and middle-income countries. Moreover, compared to low- and middle-income countries, Canada had a larger weighted PAF for midlife obesity.

### By Sex and Age groups

Prevalence of the most common risk factors differed across sexes (Table 4 and Figure S4 in Appendix). Among women, 80.0% had physical inactivity and 20.8% had depression, as compared to 72.8% and 11.8% in men. The prevalence of less education and sleep disturbances were similar between sexes (p>0.05). The prevalence of hearing loss, traumatic brain injury, hypertension, excessive alcohol use, diabetes, and social isolation was higher in men than women (p<0.001).

**Table 4:** Prevalence and weighted population attributable fraction of 12 potentially modifiable risk factors by four age groups and sex in Canada

	**Overall**		**Age group**									**Sex**				
			**45–54**		**55–64**		**65–74**		**75–85**			**Women**		**Men**		
**Risk factors**	**Prev (%)**	**wPAF (%)**	**Prev (%)**	**wPAF (%)**	**Prev (%)**	**wPAF (%)**	**Prev (%)**	**wPAF (%)**	**Prev (%)**	**wPAF (%)**	p*	**Prev (%)**	**wPAF (%)**	**Prev (%)**	**wPAF (%)**	p*
Less education	12.6	2.7	7.2	1.9	12.5	2.6	16.8	3.9	24.7	5.8	<0.001	12.1	2.8	13.2	2.8	0.3
Hearing loss	33.2	8.7	13.5	4.9	28.5	7.7	54.2	14.1	81.2	19.0	<0.001	29.6	8.7	37.1	9.6	<0.001
Traumatic brain injury	13.9	3.8	15.3	5.0	14.4	3.9	13.0	4.0	8.9	3.0	<0.001	10.3	3.1	17.8	4.8	<0.001
Hypertension	37.0	6.9	21.5	5.2	38.8	7.1	53.2	10.4	59.8	11.9	<0.001	35.4	7.2	38.8	7.3	<0.001
Excessive alcohol use	11.5	0.9	10.9	1.0	12.4	0.9	12.4	1.0	9.8	0.9	<0.001	6.4	0.5	17.1	1.3	<0.001
Obesity	30.4	5.8	27.3	6.4	34.5	6.4	32.9	7.1	26.0	6.1	<0.001	30.1	6.3	30.7	6.0	0.02
Smoking	10.7	2.3	13.7	3.5	11.9	2.5	6.4	1.6	3.9	1.0	<0.001	10.7	2.5	10.7	2.3	0.04
Depression	16.5	4.9	16.4	5.9	20.6	5.9	14.3	4.9	9.0	3.4	<0.001	20.8	6.5	11.8	3.7	<0.001
Social isolation	1.6	0.4	1.9	0.5	1.4	0.3	0.9	0.2	1.8	0.5	0.003	1.0	0.2	2.2	0.5	<0.001
Physical inactivity	76.6	8.9	71.6	10.2	76.8	8.8	79.8	10.4	87.8	11.7	<0.001	80.0	10.0	72.8	8.7	<0.001
Diabetes	8.5	1.6	4.3	1.0	10.3	1.8	12.3	2.5	12.1	2.6	<0.001	7.2	1.4	10.0	1.8	<0.001
Sleep disturbances	43.0	3.0	43.0	3.6	45.3	3.1	39.7	3.2	41.7	3.5	0.004	42.2	3.2	43.7	3.1	0.1
Combined PAF		49.7		49.1		51.1		63.4		69.3			52.4		51.9	

Prev, Prevalence; wPAF, Weighted population attributable fraction; * Chi-square test comparing the prevalence of risk factor by age groups or sex; Chi-square test was weighted with the CLSA analytic weight

Overall, the 12 risk factors accounted for 52.4% (95% CI 32.5% to 68.7%) of dementia among women and 51.9% (95% CI 32.2% to 68.0%) among men (Table 4 and Figure S5 in Appendix). The weighted PAF of hearing loss, traumatic brain injury, and excessive alcohol use was higher in men, whereas the weighted PAF of depression and physical inactivity was higher in women.

Prevalence and weighted PAF also varied across age groups (Table 4 and Figure S6 in Appendix). From age 45–54 to 75–85 years, prevalence increased from 13.5% to 81.2% for hearing loss, 7.2% to 24.7% for less education, 21.5% to 59.8% for hypertension, 71.6% to 87.8% for physical inactivity, and 4.3% to 12.1% for diabetes. The highest prevalence of traumatic brain injury (15.3%) and smoking (13.7%) was observed at age 45–54 years, which declined after age 55 years and dropped to 8.9% and 3.9% at age 75–85 years, respectively. Depression prevalence increased from 16.4% in age 45–54 to 20.6% in 55–64 years, then decreased to 9.0% in 75–85 years.

The combined PAF of all 12 risk factors increased with age (Figure S5 in Appendix). The combined PAF was 49.1% (95% CI 30.5% to 65.5%) in age 45–54 years, 51.1% (95% CI 31.4% to 67.6%) in 55–64 years, 63.4% (95% CI 40.7% to 79.4%) in 65–74 years, and 68.2% (95% CI 46.2% to 83.9%) in 75–85 years. Physical inactivity had the highest weighted PAF among ages 45–54 years (10.2%, 95% CI 6.6% to 13.2%) and 55–64 years (8.8%, 95% CI 5.7% to 11.4%), while hearing loss had the largest weighted PAF among ages 65–74 years (14.1%, 95% CI 9.0% to 17.3%) and 75–85 years (19.0%, 95% CI 13.4% to 21.6%). The estimated communality and unadjusted PAFs by age groups and sex are shown in Table S3–S4 in Appendix.

### Sensitivity Analyses

Operationalizing risk factors with different definitions increased the prevalence and PAF, and also changed the rank of risk factors with the largest PAF (Table S5–S7 in Appendix). The prevalence increased to 16% for depression, 29% for excessive alcohol use, and 42% for social isolation. The largest change was observed with social isolation, and its prevalence increased from 1.6% to 42%. The combined PAF increased to 53.2% (95% CI 33.9% to 68.9%). Later life social isolation became the risk factor with the second largest PAF. In older age groups, social isolation had larger PAF than obesity.

## Discussion

Our results suggest that up to 49% of dementia cases in Canada may be attributable to 12 modifiable risk factors. To our knowledge, this is the first study estimating the potential population impact of 12 modifiable risk factors for dementia in this country and to include sleep disturbances in a life-course model with participants as young as 45 years. The predominant contributors were later life physical inactivity, midlife hearing loss, midlife obesity, and midlife hypertension. In contrast, later life smoking, midlife excessive alcohol use, and later life social isolation had substantially less contribution. Finally, we observed a similar prevention potential in men and women.

Over 80% of Canadians were not meeting physical activity guidelines, and nearly 40% of Canadians had impaired sleeping. Furthermore, 1 in 3 Canadians were obese or had hypertension, and 1 in 5 Canadians showed hearing loss. The estimated prevalence of obesity, physical inactivity, diabetes, and sleep disturbances was similar to previous estimates, but our results showed a higher prevalence of less education and hypertension, and a lower prevalence of smoking and hearing loss (27, 28).

Despite an unclear underlying mechanism between hearing loss and dementia, a meta-analysis of 8 longitudinal studies found a 19% lower risk of cognitive decline among individuals with corrected versus uncorrected hearing loss (29). These findings suggest that dementia prevention programs and primary health care providers should consider encouraging hearing tests and hearing aid use. Compared to other lifestyle strategies, addressing hearing loss faces challenges related to stigma and affordability of hearing aids (30). Given its high PAF and associated barriers, there is a pressing need to enhance hearing health care besides promoting patient education.

Sleep disturbances are an emerging potential contributor to dementia, and the high prevalence we found could be translated to brain health benefits at the population level. For instance, non-pharmacological interventions for sleep disturbances or disorders include sleep hygiene education, physical activity, and, in some cases, bright light therapy (31). However their effectiveness in preventing cognitive impairment requires further study due to potential reverse causation (32). Periodically screening sleep impairment in practice will help identify and intervene undiagnosed sleep impairment.

The combined PAF of the nine risk factors that are available across all studies was 33.2% globally and ranged between 33.2% and 55.9% among studied countries. The proportion of potentially preventable dementia cases attributable to these nine risk factors in Canada was similar to studies from low- and middle-income countries, but higher than those from other high-income countries. Latin America reported the highest combined PAF, using data from 6 Latin American countries (9).

The risk factor with the highest weighted PAF was less education in low- and middle-income countries, while it was physical inactivity and obesity in high-income countries, suggesting a sociocultural gradient in dementia risk factors. Notably, risk factor profiles varied even among high-income countries, highlighting the uniqueness of the Canadian population, which could inform policymakers in Canada. For instance, despite cultural similarities between Canada and USA, the prevalence of hearing loss, depression, and alcohol use was 2 to 4 times higher in Canada, while USA had 1.5 times or more higher prevalence of obesity, diabetes, hypertension, and social isolation (7). Interestingly, although obesity was more prevalent in USA, Canada had a higher prevalence of physical inactivity. Despite geographical distance, the risk factor profile of Canada was fairly similar to Australia and Denmark. This underpins the importance of identifying population-specific risk factor profiles to inform researchers and policymakers on developing dementia risk reduction strategies. International differences in risk factor prevalence could also reflect healthcare and policy contexts. For instance, the relatively lower prevalence of less education and smoking in high-income countries might be explained by the implementation of governmental compulsory education (33) and tobacco control policies (34).

Our age-specific analysis found that physical inactivity, less education, and risk factors related to chronic diseases, such as hearing loss, hypertension, and diabetes, gradually increase with age. Similar findings were observed in a study conducted in Chilean population (35). The decreasing trend observed for traumatic brain injury, smoking, and excessive alcohol use may imply a decline in risk-taking with aging (36).

As with other studies ascertaining sex-specific estimates (35, 37, 38), the prevalence of traumatic brain injury, hearing loss and excessive alcohol use were higher in men while depression and physical inactivity were more prevalent in women. Contrary to our results, sex differences in smoking were observed in previous studies (35, 37, 38). Although we obtained a similar potential for dementia prevention in men and women, the observed difference in risk factor profile is important to inform public policy to focus on addressing early risk behaviours, particularly in men.

Importantly, the difference in the risk factor profile we observed by age groups and sex highlights the importance of tailoring national dementia prevention programs and strategies. For instance, dementia prevention programs for middle-aged adults should include education on risk-taking behaviours, while the focus could shift to better management of hearing loss, hypertension, and diabetes in older adults. The programs for older adults should prioritize delivering group exercise or recreational programs. The dementia prevention efforts could be further enhanced by focusing on addressing depression in women and hearing loss in men.

The World Health Organization’s Global Action Plan as well as Canada’s National Dementia Strategy includes the promotion of a healthy lifestyle to reduce dementia risk, such as initiatives and programs to prevent and manage non-communicable diseases (27, 39, 40). However, the observed high prevalence of physical inactivity highlights the need to advocate and implement moderate-to-vigorous exercise programs for older adults to help them meet physical activity targets. For instance, aerobic and resistance exercises have been shown to improve cognition in older adults and can be delivered in exercise programs (41, 42). Innovative approaches including exercises and effective coaching delivered by digital apps and platforms could help tackle this issue (16, 43). Importantly, the high prevalence of hypertension and obesity further underscores the opportunity to improve both vascular and brain health.

The primary strength of our study is the use of CLSA data, the largest and well-characterized cohort study in Canada. Its large sample of over 30,000 participants allowed us to explore the risk factor distributions from midlife to later life. Specifically, we showed dementia risk factor distributions and their PAF across 4 age groups beginning at age 45. In addition, as a single data source was used to measure all 12 modifiable risk factors, we were able to avoid making assumptions of communality for any variables, which can undermine the precision of the analyses.

There are several limitations. First, some risk factors were defined based on self-reported data and misclassification of risk factors may impact the estimated PAF. For example, depression and sleep disturbances were classified based on self-reported measures, although using valid instruments. Physical inactivity excluded light activities. However, because our sensitivity analyses produced higher estimates of PAF than our main analysis, our estimates probably underestimate PAF. Second, Levin’s formula assumes no confounding and it produces a biased estimate of PAF when adjusted risk ratio is used. Since covariate adjustments tend to reduce risk ratio estimates, our obtained PAF may be underestimated (44). Third, the PAF calculation relies on risk ratio and prevalence which are estimated at an aggregated level and likely to be dependent on participant characteristics such as ethnicity. While Canada is an ethnoculturally diverse country, with 70% of Canadians reported being White, our sample was composed by largely highly educated White Canadians, living in urban settings (45). This suggests that the obtained prevalence may not accurately reflect risk factor prevalence in other population groups, limiting generalizability. For the age and sex-specific PAF calculation, the risk ratio used was from the life course-specific measures and may not accurately represent other age groups or sexes. The Norton’s formula we used to estimate the combined PAF assumes the independence of risk factors by weighting risk factor overlapping (20, 46). However, this approach of adjusting for risk factor inter-relationship to combine PAF may not fully account for confounding, interactions among risk factors, and its multifactorial nature. While the new approach to calculate the combined PAF without assuming independence by robustly accounting for the complexity and inter-relationship of risk factors has been proposed, our results can be seen as conservative as the new approach presented a higher estimation of combined PAF (46). An important limitation in the interpretation of PAF is it relies on assumptions of causality between risk factors and dementia, such as hypertension which fulfills the causality criteria. However, the causality has not been fully established for some risk factors including sleep disturbances, which may increase the risk of dementia but can also be part of the pathophysiological process of dementia (32). Although diet is recognized as an important dementia risk factor (47), it was not included due to the complexities of analyzing diet. Lastly, while most of the risk factors had similar definitions, caution is needed in comparing the prevalence and PAF across studies.

Our results have critical implications for public health and policymakers by demonstrating the prevalence and the potential population impact of dementia modifiable risk factors that can be targeted in national strategies or intervention programs, such as promotion of physical activity, and by helping in prioritizing the most beneficial risk factors to be combated.

Implications for healthcare professionals included awareness of potentially reversible dementia risk factors that could be screened and treated from midlife. Interestingly, several of the risk factors identified are also critical for cardiovascular health, therefore their prevention and treatment can theoretically improve both cardiovascular and brain health.

Although today the β-amyloid-targeting therapies in early symptomatic Alzheimer’s disease is showing promising results (48, 49) there is emerging evidence that the beneficial effect of lifestyle interventions are regardless of β-amyloid pathology burden (50). Multidomain intervention trials targeting modifiable lifestyle risk factors including the seminal FINGER trial (51) and subgroup analyses of other large prevention trials (52, 53) have started to show positive results in improving cognition. The FINGER model is being adapted and tested globally within the World-Wide FINGERS network of multidomain trials for dementia risk reduction (14), including the SYNERGIC trials and Brain Health PRO in Canada, part of the Canadian Therapeutic Platform Trial for Multidomain Interventions to Prevent Dementia (CAN-THUMBS UP) initiative (16, 41, 43). Additionally, combining lifestyle intervention with disease-modifying therapy will offer an even better opportunity to effectively and precisely manage and prevent dementia based on patient’s risk profiles, as demonstrated in diabetes management (54), and multidomain lifestyle-based interventions combined with repurposed drugs (metformin) are now being tested in Europe (55). Importantly, not all older adults at risk of dementia will be candidates for emerging disease-modifying therapy due to the therapies contraindications and limited generalizability of the current trial results, and therefore lifestyle interventions may be more applicable (56). Analyses of the most prevalent combinations of risk factors and risk factor clustering would allow the development of impactful personalized prevention programs.

In conclusion, we determined that nearly up to 50% of dementia cases in Canada are attributable to 12 modifiable risk factors across the life span. The most prominent modifiable risk factors were later life physical inactivity, midlife obesity, midlife hypertension, and midlife hearing loss, whereas midlife excessive alcohol use, later life smoking, and later life social isolation had substantially less contribution.

## Appendix for

Potentially Modifiable Dementia Risk Factors in Canada: An Analysis of Canadian Longitudinal Study on Aging with a Multi-Country Comparison

## Notes

*Ethical standards:* All participants of CLSA study provided informed consent.

## References

[1] GBD Dementia Forecasting Collaborators. Estimation of the global prevalence of dementia in 2019 and forecasted prevalence in 2050: an analysis for the Global Burden of Disease Study 2019. Lancet Public Health. Feb 2022;7(2):e105–e125. doi:10.1016/S2468-2667(21)00249-8 34998485 10.1016/S2468-2667(21)00249-8 PMC8810394

[2] Alzheimer Society Canada. Navigating the path forward for dementia in Canada: The Landmark Study Report #1. 2022.

[3] Baumgart M, Snyder HM, Carrillo MC, Fazio S, Kim H, Johns H. Summary of the evidence on modifiable risk factors for cognitive decline and dementia: A population-based perspective. Alzheimers Dement. Jun 2015;11(6):718–26. doi:10.1016/j.jalz.2015.05.016 26045020 10.1016/j.jalz.2015.05.016

[4] Kivipelto M, Mangialasche F, Ngandu T. Lifestyle interventions to prevent cognitive impairment, dementia and Alzheimer disease. Nat Rev Neurol. Nov 2018;14(11):653–666. doi:10.1038/s41582-018-0070-3 30291317 10.1038/s41582-018-0070-3

[5] Livingston G, Huntley J, Sommerlad A, et al. Dementia prevention, intervention, and care: 2020 report of the Lancet Commission. Lancet. Aug 8 2020;396(10248):413–446. doi:10.1016/S0140-6736(20)30367-6 32738937 10.1016/S0140-6736(20)30367-6 PMC7392084

[6] Livingston G, Sommerlad A, Orgeta V, et al. Dementia prevention, intervention, and care. Lancet. Dec 16 2017;390(10113):2673–2734. doi:10.1016/S0140-6736(17)31363-6 28735855 10.1016/S0140-6736(17)31363-6

[7] Lee M, Whitsel E, Avery C, et al. Variation in Population Attributable Fraction of Dementia Associated With Potentially Modifiable Risk Factors by Race and Ethnicity in the US. JAMA Netw Open. Jul 1 2022;5(7):e2219672. doi:10.1001/jamanetworkopen.2022.19672 35793088 10.1001/jamanetworkopen.2022.19672 PMC9260480

[8] Ma’u E, Cullum S, Cheung G, Livingston G, Mukadam N. Differences in the potential for dementia prevention between major ethnic groups within one country: A cross sectional analysis of population attributable fraction of potentially modifiable risk factors in New Zealand. Lancet Reg Health West Pac. Aug 2021;13:100191. doi:10.1016/j.lanwpc.2021.100191 34527984 10.1016/j.lanwpc.2021.100191 PMC8358157

[9] Mukadam N, Sommerlad A, Huntley J, Livingston G. Population attributable fractions for risk factors for dementia in low-income and middle-income countries: an analysis using cross-sectional survey data. Lancet Glob Health. May 2019;7(5):e596–e603. doi:10.1016/S2214-109X(19)30074-9 31000129 10.1016/S2214-109X(19)30074-9 PMC7617123

[10] See RS, Thompson F, Russell S, et al. Potentially modifiable dementia risk factors in all Australians and within population groups: an analysis using cross-sectional survey data. Lancet Public Health. Sep 2023;8(9):e717–e725. doi:10.1016/S2468-2667(23)00146-9 37633680 10.1016/S2468-2667(23)00146-9

[11] Suemoto CK, Mukadam N, Brucki SMD, et al. Risk factors for dementia in Brazil: Differences by region and race. Alzheimers Dement. May 2023;19(5):1849–1857. doi:10.1002/alz.12820 36326095 10.1002/alz.12820

[12] Jorgensen K, Nielsen TR, Nielsen A, Waldemar G. Potential for prevention of dementia in Denmark. Alzheimers Dement. Oct 2023;19(10):4590–4598. doi:10.1002/alz.13030 36933232 10.1002/alz.13030

[13] Flanagan A, Dunning, J., Wong, I., Brierley, A., MacDonald, BJ., Sinha, SK. Enabling a More Promising Future for Long-Term Care in Canada. 2023.

[14] Kivipelto M, Mangialasche F, Snyder HM, et al. World-Wide FINGERS Network: A global approach to risk reduction and prevention of dementia. Alzheimers Dement. Jul 2020;16(7):1078–1094. doi:10.1002/alz.12123 32627328 10.1002/alz.12123 PMC9527644

[15] Montero-Odasso M, Ismail Z, Livingston G. One third of dementia cases can be prevented within the next 25 years by tackling risk factors. The case “for” and “against”. Alzheimers Res Ther. Jul 8 2020;12(1):81. doi:10.1186/s13195-020-00646-x 32641088 10.1186/s13195-020-00646-x PMC7346354

[16] Feldman HH, Belleville S, Nygaard HB, et al. Protocol for the Brain Health Support Program Study of the Canadian Therapeutic Platform Trial for Multidomain Interventions to Prevent Dementia (CAN-THUMBS UP): A Prospective 12-Month Intervention Study. J Prev Alzheimers Dis. 2023;10(4):875–885. doi:10.14283/jpad.2023.65 37874110 10.14283/jpad.2023.65 PMC10258470

[17] Chen H, Cao Y, Ma Y, Xu W, Zong G, Yuan C. Age- and sex-specific modifiable risk factor profiles of dementia: evidence from the UK Biobank. Eur J Epidemiol. Jan 2023;38(1):83–93. doi:10.1007/s10654-022-00952-8 36593335 10.1007/s10654-022-00952-8

[18] Raina PS, Wolfson C, Kirkland SA, et al. The Canadian longitudinal study on aging (CLSA). Can J Aging. Sep 2009;28(3):221–9. doi:10.1017/S0714980809990055 19860977 10.1017/S0714980809990055

[19] Barnes DE, Yaffe K. The projected effect of risk factor reduction on Alzheimer’s disease prevalence. Lancet Neurol. Sep 2011;10(9):819–28. doi:10.1016/S1474-4422(11)70072-2 21775213 10.1016/S1474-4422(11)70072-2 PMC3647614

[20] Norton S, Matthews FE, Barnes DE, Yaffe K, Brayne C. Potential for primary prevention of Alzheimer’s disease: an analysis of population-based data. Lancet Neurol. Aug 2014;13(8):788–94. doi:10.1016/S1474-4422(14)70136-X 25030513 10.1016/S1474-4422(14)70136-X

[21] World Health Organization. World Report on Hearing. 2021. https://www.who.int/publications-detail-redirect/world-report-on-hearing

[22] National Health Services England. Alcohl units. https://www.nhs.uk/live-well/alcohol-advice/calculating-alcohol-units/

[23] Shi L, Chen SJ, Ma MY, et al. Sleep disturbances increase the risk of dementia: A systematic review and meta-analysis. Sleep Med Rev. Aug 2018;40:4–16. doi:10.1016/j.smrv.2017.06.010 28890168 10.1016/j.smrv.2017.06.010

[24] Canadian Longitudinal Study on Aging. CLSA Technical Report 2023: Sampling and Computation of Response Rates and Sample Weights for the Tracking (Telephone Interview) Participants and Comprehensive Participants. 2023. https://www.clsa-elcv.ca/doc/5130

[25] Paradis C, Butt P, Shield K, et al. Canada’s Guidance on Alcohol and Health: Final Report. 2023.

[26] R A Language and Environment for Statistical Computing. R Foundation for Statistical Computing; 2023.

[27] Public Health Agency of Canada. A dementia strategy for Canada: 2021 Annual report. 2021.

[28] Ramage-Morin PL., Banks R., Pineault D., Atrach M., H. G. Hypertension associated with hearing health problems among Canadian adults aged 19 to 79 years. 2021. Health Reports. 10.25318/82-003-x202101000002-eng 34669323

[29] Yeo CD, Yeom SW, You YS, Kim JS, Lee EJ. Association of sudden sensorineural hearing loss with increased risk of insomnia: a nationwide population-based cohort study. J Clin Sleep Med. May 1 2022;18(5):1335–1342. doi:10.5664/jcsm.9864 34978279 10.5664/jcsm.9864 PMC9059585

[30] Jenstad L, Moon J. Systematic Review of Barriers and Facilitators to Hearing Aid Uptake in Older Adults. Audiol Res. May 10 2011;1(1):e25. doi:10.4081/audiores.2011.e25 26557310 10.4081/audiores.2011.e25 PMC4627148

[31] Zee PC, Vitiello MV. Circadian Rhythm Sleep Disorder: Irregular Sleep Wake Rhythm Type. Sleep Med Clin. Jun 1 2009;4(2):213–218. doi:10.1016/j.jsmc.2009.01.009 20160950 10.1016/j.jsmc.2009.01.009 PMC2768129

[32] Wennberg AMV, Wu MN, Rosenberg PB, Spira AP. Sleep Disturbance, Cognitive Decline, and Dementia: A Review. Semin Neurol. Aug 2017;37(4):395–406. doi:10.1055/s-0037-1604351 28837986 10.1055/s-0037-1604351 PMC5910033

[33] Besche-Truthe F. The global trajectories of compulsory education: clustering sequences of policy development. Global Pathways to Education Global Dynamics of Social Policy. Palgrave Macmillan, Cham.; 2021.

[34] Anderson CL, Becher H, Winkler V. Tobacco Control Progress in Low and Middle Income Countries in Comparison to High Income Countries. Int J Environ Res Public Health. Oct 24 2016;13(10)doi:10.3390/ijerph13101039 10.3390/ijerph13101039 PMC5086778 27783060

[35] Vergara RC, Zitko P, Slachevsky A, San Martin C, Delgado C. Population attributable fraction of modifiable risk factors for dementia in Chile. Alzheimers Dement (Amst). 2022;14(1):e12273. doi:10.1002/dad2.12273 35229017 10.1002/dad2.12273 PMC8864720

[36] Rolison JJ, Hanoch Y, Wood S, Liu PJ. Risk-taking differences across the adult life span: a question of age and domain. J Gerontol B Psychol Sci Soc Sci. Nov 2014;69(6):870–80. doi:10.1093/geronb/gbt081 24149517 10.1093/geronb/gbt081

[37] Borelli WV, Leotti VB, Strelow MZ, Chaves MLF, Castilhos RM. Preventable risk factors of dementia: Population attributable fractions in a Brazilian population-based study. Lancet Reg Health Am. Jul 2022;11:100256. doi:10.1016/j.lana.2022.100256 36778926 10.1016/j.lana.2022.100256 PMC9903643

[38] Nianogo RA, Rosenwohl-Mack A, Yaffe K, Carrasco A, Hoffmann CM, Barnes DE. Risk Factors Associated With Alzheimer Disease and Related Dementias by Sex and Race and Ethnicity in the US. JAMA Neurol. Jun 1 2022;79(6):584–591. doi:10.1001/jamaneurol.2022.0976 35532912 10.1001/jamaneurol.2022.0976 PMC9086930

[39] Public Health Agency of Canada. A dementia strategy for Canada. 2019.

[40] World Health Organization. Global action plan on the public health response to dementia 2017–2025. 2017.

[41] Montero-Odasso M, Zou G, Speechley M, et al. Effects of Exercise Alone or Combined With Cognitive Training and Vitamin D Supplementation to Improve Cognition in Adults With Mild Cognitive Impairment: A Randomized Clinical Trial. JAMA Netw Open. Jul 3 2023;6(7):e2324465. doi:10.1001/jamanetworkopen.2023.24465 37471089 10.1001/jamanetworkopen.2023.24465 PMC10359965

[42] Northey JM, Cherbuin N, Pumpa KL, Smee DJ, Rattray B. Exercise interventions for cognitive function in adults older than 50: a systematic review with meta-analysis. Br J Sports Med. Feb 2018;52(3):154–160. doi:10.1136/bjsports-2016-096587 28438770 10.1136/bjsports-2016-096587

[43] McGibbon C, Jarrett P, Handrigan G, et al. Protocol for SYNchronising Exercises, Remedies in GaIt and Cognition at Home (SYNERGIC@Home): feasibility of a home-based double-blind randomised controlled trial to improve gait and cognition in individuals at risk for dementia. BMJ Open. Mar 31 2022;12(3):e059988. doi:10.1136/bmjopen-2021-059988 35361653 10.1136/bmjopen-2021-059988 PMC8971768

[44] Darrow LA, Steenland NK. Confounding and bias in the attributable fraction. Epidemiology. Jan 2011;22(1):53–8. doi:10.1097/EDE.0b013e3181fce49b 20975564 10.1097/EDE.0b013e3181fce49b

[45] Statistics Canada. The Canadian census: A rich portrait of the country’s religious and ethnocultural diversity. 2022. https://www150.statcan.gc.ca/n1/daily-quotidien/221026/dq221026b-eng.htm

[46] Welberry HJ, Tisdell CC, Huque MH, Jorm LR. Have We Been Underestimating Modifiable Dementia Risk? An Alternative Approach for Calculating the Combined Population Attributable Fraction for Modifiable Dementia Risk Factors. Am J Epidemiol. Oct 10 2023;192(10):1763–1771. doi:10.1093/aje/kwad138 37326043 10.1093/aje/kwad138 PMC10558200

[47] World Health Organization. Risk reduction of cognitive decline and dementia: WHO guidelines. 2019. 31219687

[48] Sims JR, Zimmer JA, Evans CD, et al. Donanemab in Early Symptomatic Alzheimer Disease: The TRAILBLAZER-ALZ 2 Randomized Clinical Trial. JAMA. Jul 17 2023;doi:10.1001/jama.2023.13239 10.1001/jama.2023.13239 PMC10352931 37459141

[49] van Dyck CH, Swanson CJ, Aisen P, et al. Lecanemab in Early Alzheimer’s Disease. N Engl J Med. Jan 5 2023;388(1):9–21. doi:10.1056/NEJMoa2212948 36449413 10.1056/NEJMoa2212948

[50] Dhana K, Agarwal P, James BD, et al. Healthy Lifestyle and Cognition in Older Adults With Common Neuropathologies of Dementia. JAMA Neurol. Feb 5 2024;doi:10.1001/jamaneurol.2023.5491 10.1001/jamaneurol.2023.5491 PMC10845037 38315471

[51] Ngandu T, Lehtisalo J, Solomon A, et al. A 2 year multidomain intervention of diet, exercise, cognitive training, and vascular risk monitoring versus control to prevent cognitive decline in at-risk elderly people (FINGER): a randomised controlled trial. Lancet. Jun 6 2015;385(9984):2255–63. doi:10.1016/S0140-6736(15)60461-5 25771249 10.1016/S0140-6736(15)60461-5

[52] Andrieu S, Guyonnet S, Coley N, et al. Effect of long-term omega 3 polyunsaturated fatty acid supplementation with or without multidomain intervention on cognitive function in elderly adults with memory complaints (MAPT): a randomised, placebo-controlled trial. Lancet Neurol. May 2017;16(5):377–389. doi:10.1016/S1474-4422(17)30040-6 28359749 10.1016/S1474-4422(17)30040-6

[53] Moll van Charante EP, Richard E, Eurelings LS, et al. Effectiveness of a 6-year multidomain vascular care intervention to prevent dementia (preDIVA): a cluster-randomised controlled trial. Lancet. Aug 20 2016;388(10046):797–805. doi:10.1016/S0140-6736(16)30950-3 27474376 10.1016/S0140-6736(16)30950-3

[54] Torgerson JS, Hauptman J, Boldrin MN, Sjostrom L. XENical in the prevention of diabetes in obese subjects (XENDOS) study: a randomized study of orlistat as an adjunct to lifestyle changes for the prevention of type 2 diabetes in obese patients. Diabetes Care. Jan 2004;27(1):155–61. doi:10.2337/diacare.27.1.155 14693982 10.2337/diacare.27.1.155

[55] Barbera M, Lehtisalo J, Perera D, et al. A multimodal precision-prevention approach combining lifestyle intervention with metformin repurposing to prevent cognitive impairment and disability: the MET-FINGER randomised controlled trial protocol. Alzheimers Res Ther. Jan 31 2024;16(1):23. doi:10.1186/s13195-023-01355-x 38297399 10.1186/s13195-023-01355-x PMC10829308

[56] Manly JJ, Deters KD. Donanemab for Alzheimer Disease-Who Benefits and Who Is Harmed? JAMA. Aug 8 2023;330(6):510–511. doi:10.1001/jama.2023.11704 10.1001/jama.2023.11704 37459138

