# Supplementary material for: Potentially Modifiable Dementia Risk Factors in Canada: An Analysis of Canadian Longitudinal Study on Aging with a Multi-Country Comparison
Source: J Prev Alzheimers Dis. 2024 Jun 7;11(5):1490–9. doi: 10.14283/jpad.2024.105 (PMC11436427; doi:10.14283/jpad.2024.105)
Supplement: Supplementary file 1 — Potentially Modifiable Dementia Risk Factors in Canada: An Analysis of Canadian Longitudinal Study on Aging with a Multi-Country Comparison [file 42414_2024_105_MOESM1_ESM.docx]

**Appendix**

for

S. Son, M. Speechley, G.Y. Zou, M. Kivipelto, F. Mangialasche, H.H. Feldman, H. Chertkow, S. Belleville, H. Nygaard, V. Hachinski, F. Pieruccini-Faria, M. Montero-Odasso. **Potentially Modifiable Dementia Risk Factors in Canada: An Analysis of Canadian Longitudinal Study on Aging with a Multi-Country Comparison**

**Table of Contents**

**Figure S1. CLSA study design overview**

**Table S1. Sleep disturbances classification**

**Table S2. PAF Calculation**

**Figure S2. Prevalence of modifiable risk factors for dementia across the world**

**Figure S3. Weighted PAF of modifiable risk factors for dementia across the world**

**Table S3. PAF for 12 modifiable risk factors for dementia in Canada by sex**

**Figure S4. Prevalence of modifiable risk factors for dementia in Canada by sex**

**Figure S5. Weighted PAF of modifiable risk factors for dementia in Canada by four age groups and sex**

**Table S4. PAF for 12 modifiable risk factors for dementia in Canada by four age groups**

**Figure S6. Prevalence of modifiable risk factors for dementia in Canada by four age groups**

**Table S5. Sensitivity analysis for life-course model**

**Table S6. Sensitivity analysis by four age groups**

**Table S7. Sensitivity analysis by sex**

**Figure S1. CLSA Study design overview**

Adapted from Raina et al. (2019)^1^

**Table S1. Sleep disturbances classification**

| Type | Definition |
| --- | --- |
| Poor sleep quality | *“How satisfied or dissatisfied are you with your current sleep pattern?”*   - Dissatisfaction: Very dissatisfied, dissatisfied - Satisfaction: Very satisfied, satisfied, neutral |
| Insomnia symptom | 1. **Difficulty initiating sleep more than 3 times a week which significantly interfere with daily function:**   *“Over the last month, how often did it take you more than 30 minutes to fall asleep?”*   - Yes: 3-5 times/week, 6-7 times/week - No: Never, <1/week, Once or twice/week   AND  *“To what extent do you consider your problem falling asleep to interfere with your daily functioning”*   - Yes: Much, Very much - No: Not at all, A little, Somewhat   **Or (ii) Trouble maintaining sleep more than 3 times a week which significantly interfere with daily function:**  *“Over the last month, how often did you wake in the middle morning and found it difficult to fall asleep again?”*   - Yes: 3-5 times/week, 6-7 times/week - No: Never, <1/week, Once or twice/week   AND  *“To what extent do you consider your problem staying asleep to interfere with your daily functioning”*   - Yes: Much, Very much - No: Not at all, A little, Somewhat |
| Sleep apnoea | *“Has anyone ever observed you stop breathing in your sleep?”*   - Yes - No |
| Daytime sleepiness | *“Over the last month, how often do you find it difficult to stay awake during your normal waking hours when you want to?*   - Yes: 3-5 times/week, 6-7 times/week - No: Never, <1/week, Once or twice/week |
| Restless leg syndrome | 1. **Uncomfortable feeling in leg:**   *“Do you have, or have you sometimes experienced, recurrent, uncomfortable feelings or sensations in your legs while sitting or lying down?”*   - Yes - No   **And (ii) Urge to move leg:**  *“Do you have, or have you sometimes experienced, a recurrent need or urge to move your legs while sitting or lying down?”*   - Yes - No   **And (iii) uncomfortable feeling/urge to move leg more than 3 times per week:**  *“Over the last month, how many times (per week, on average) have you experienced these uncomfortable feelings or urge to move?”*   - Yes: Three or four times, More than four times - Less than once, Once or twice   **And (iv) Disappear when moving around**  *“Do these uncomfortable feelings or sensations in your legs, or the urge to move, disappear/improve when you are active or moving around?”*   - Yes - No   **And (v) Worse in the evening**  *“Are these uncomfortable feelings, or this urge to move, worse in the evening or at night compared with the morning?”*   - Yes - No |

**Table S2. PAF Calculation**

| **Step 1** | The PAF of each risk factor was calculated using Levin’s formula:  $\text{PAF = }\text{P}_{\text{e}}\frac{\text{(R}\text{R}_{\text{e}}\text{-1)}}{\text{1+}\text{P}_{\text{e}}\text{(R}\text{R}_{\text{e}}\text{-1)}}$  where P_e_ is the prevalence of risk factor, RR_e_ is relative risk of risk factor |
| --- | --- |
| **Step 2** | Principal component analysis was performed on the tetrachoric correlation matrix to produce weight (w). Weight was calculated by subtracting the communality (h^2^) from 1:  w = 1 – h^2^. |
| **Step 3** | The overall weighted PAF (wPAF) was obtained by:  $\text{Combined }\text{wPA}\text{F}\text{ }\text{= 1 - [}\left( \text{1 - }\text{ w}_{\text{1 }}\text{* PA}\text{F}_{\text{1}} \right)\text{ × … × }\left( \text{1 - }\text{w}_{\text{12}}\text{ * PA}\text{F}_{\text{12}} \right)\text{]}$ |
| **Step 4** | Individual weighted PAF was determined as:  $w\text{PA}\text{F}_{\text{i }}\text{ = }\frac{\text{PA}\text{F}_{\text{i}}}{\sum_{i} PAF_{i}}\text{ × Combined wPA}\text{F}$ |

**Figure S2. Prevalence of modifiable risk factors for dementia across the world**

**^*^** Risk factors available across all studies

**Figure S3. Weighted PAF of modifiable risk factors for dementia across the world**

**^*^** Risk factors available across all studies

**Table S3. PAF for 12 modifiable risk factors for dementia in Canada by sex**

|  | Women |  |  |  |  | Men |  |  |  |
| --- | --- | --- | --- | --- | --- | --- | --- | --- | --- |
| Risk factors | Communality (%) | Prevalence (%) | Unweighted PAF (%) | Weighted PAF (%) |  | Communality (%) | Prevalence (%) | Unweighted PAF (%) | Weighted PAF (%) |
| Less education | 56.9 | 12.1 | 6.8 | 2.8 (1.7, 3.8) |  | 59.4 | 13.2 | 7.3 | 2.8 (1.7, 3.8) |
| Hearing loss | 45.5 | 29.6 | 21.1 | 8.7 (5.0, 11.8) |  | 43.5 | 37.1 | 25.1 | 9.6 (5.8, 12.8) |
| Traumatic brain injury | 29.6 | 10.3 | 7.6 | 3.1 (2.3, 3.9) |  | 37.0 | 17.8 | 12.5 | 4.8 (3.6, 5.8) |
| Hypertension | 52.5 | 35.4 | 17.1 | 7.2 (3.1, 10.5) |  | 55.7 | 38.8 | 18.9 | 7.3 (3.2, 10.5) |
| Excessive alcohol | 31.0 | 6.4 | 1.27 | 0.5 (0.3, 0.7) |  | 61.1 | 17.1 | 3.3 | 1.3 (0.8, 1.6) |
| Obesity | 56.6 | 30.1 | 15.3 | 6.3 (3.9, 7.5) |  | 54.2 | 30.7 | 15.6 | 6.0 (3.8, 7.2) |
| Smoking | 50.4 | 10.7 | 6.0 | 2.5 (1.0, 4.0) |  | 63.2 | 10.7 | 6.0 | 2.3 (0.9, 3.8) |
| Depression | 50.1 | 20.8 | 15.8 | 6.5 (5.3, 7.5) |  | 49.2 | 11.8 | 9.6 | 3.7 (2.9, 4.4) |
| Social isolation | 29.3 | 1.0 | 0.6 | 0.2 (0.1, 0.3) |  | 58.2 | 2.2 | 1.3 | 0.5 (0.3, 0.6) |
| Physical inactivity | 28.7 | 80.0 | 24.2 | 10.0 (6.5, 12.7) |  | 40.7 | 72.8 | 22.6 | 8.7 (5.7, 11.2) |
| Diabetes | 57.6 | 7.2 | 3.5 | 1.4 (1.3, 1.9) |  | 53.1 | 10.0 | 4.8 | 1.8 (1.7, 2.4) |
| Sleep disturbances | 36.9 | 42.2 | 7.8 | 3.2 (1.9, 4.0) |  | 41.5 | 43.7 | 8.0 | 3.1 (1.9, 3.8) |
| Combined PAF | |  |  | 52.4 (32.5, 68.7) |  |  |  |  | 51.9 (32.2, 68.0) |

**Figure S4. Prevalence of modifiable risk factors for dementia in Canada by sex**

**Figure S5. Weighted PAF of modifiable risk factors for dementia in Canada by four age groups and sex**

**Table S4. PAF for 12 modifiable risk factors for dementia in Canada by four age groups**

| **Risk factors** | RR | Communality (%) | Prevalence (%) | Unweighted PAF (%) | Weighted PAF (%) |
| --- | --- | --- | --- | --- | --- |
| **Overall** |  |  |  |  |  |
| Less education | 1.6 | 60.5 | 12.6 | 7.0 | 2.7 (1.6, 3.7) |
| Hearing loss | 1.9 | 46.9 | 33.2 | 23.0 | 8.7 (5.1, 11.8) |
| Traumatic brain injury | 1.8 | 42.5 | 13.9 | 10.0 | 3.8 (2.8, 4.7) |
| Hypertension | 1.6 | 62.4 | 37.0 | 18.2 | 6.9 (3.0, 10.1) |
| Excessive alcohol | 1.2 | 64.2 | 11.5 | 2.3 | 0.9 (0.5, 1.1) |
| Obesity | 1.6 | 55.8 | 30.4 | 15.4 | 5.8 (3.6, 7.0) |
| Smoking | 1.6 | 58.2 | 10.7 | 6.0 | 2.3 (0.9, 3.7) |
| Depression | 1.9 | 52.0 | 16.5 | 12.9 | 4.9 (3.9, 5.8) |
| Social isolation | 1.6 | 41.3 | 1.6 | 0.9 | 0.4 (0.2, 0.5) |
| Physical inactivity | 1.4 | 37.5 | 76.6 | 23.4 | 8.9 (5.8, 11.4) |
| Diabetes | 1.5 | 54.3 | 8.5 | 4.1 | 1.6 (1.4, 2.1) |
| Sleep disturbances | 1.2 | 40.2 | 43.0 | 7.9 | 3.0 (1.8, 3.7) |
| Combined PAF |  |  |  |  | 49.7 (30.7, 65.7) |
| **45-54** |  |  |  |  |  |
| Less education | 1.6 | 52.4 | 7.2 | 4.2 | 1.9 (1.1, 2.7) |
| Hearing loss | 1.9 | 12.6 | 13.5 | 10.8 | 4.9 (2.7, 7.4) |
| Traumatic brain injury | 1.8 | 35.4 | 15.4 | 10.9 | 5 (3.8, 6.1) |
| Hypertension | 1.6 | 53.9 | 21.5 | 11.4 | 5.2 (2.2, 8.1) |
| Excessive alcohol | 1.2 | 42.3 | 10.9 | 2.1 | 1.0 (0.6, 1.3) |
| Obesity | 1.6 | 65.8 | 27.3 | 14.1 | 6.4 (4.0, 7.8) |
| Smoking | 1.6 | 60.5 | 13.7 | 7.6 | 3.5 (1.4, 5.6) |
| Depression | 1.9 | 21.7 | 16.4 | 12.9 | 5.9 (4.7, 6.9) |
| Social isolation | 1.6 | 51.6 | 1.9 | 1.1 | 0.5 (0.3, 0.7) |
| Physical inactivity | 1.4 | 34.0 | 71.6 | 22.3 | 10.2 (6.6, 13.2) |
| Diabetes | 1.5 | 60.9 | 4.3 | 2.1 | 1.0 (0.9, 1.3) |
| Sleep disturbances | 1.2 | 29.0 | 43.0 | 7.9 | 3.6 (2.2, 4.5) |
| Combined PAF |  |  |  |  | 49.1 (30.5, 65.5) |
| **55-64** |  |  |  |  |  |
| Less education | 1.6 | 60.9 | 12.5 | 7.0 | 2.6 (1.5, 3.6) |
| Hearing loss | 1.9 | 13.7 | 28.5 | 20.4 | 7.7 (4.4, 10.7) |
| Traumatic brain injury | 1.8 | 60.4 | 14.4 | 10.3 | 3.9 (2.9, 4.8) |
| Hypertension | 1.6 | 65.9 | 38.8 | 18.9 | 7.1 (3.1, 10.4) |
| Excessive alcohol | 1.2 | 64.5 | 12.4 | 2.4 | 0.9 (0.5, 1.2) |
| Obesity | 1.6 | 60.5 | 34.5 | 17.1 | 6.4 (4.0, 7.7) |
| Smoking | 1.6 | 60.8 | 11.9 | 6.6 | 2.5 (1.0, 4.1) |
| Depression | 1.9 | 55.6 | 20.6 | 15.7 | 5.9 (4.7, 6.9) |
| Social isolation | 1.6 | 36.6 | 1.4 | 0.8 | 0.3 (0.2, 0.4) |
| Physical inactivity | 1.4 | 46.2 | 76.8 | 23.5 | 8.8 (5.7, 11.4) |
| Diabetes | 1.5 | 60.5 | 10.3 | 4.9 | 1.8 (1.7, 2.5) |
| Sleep disturbances | 1.2 | 43.7 | 45.3 | 8.3 | 3.1 (1.8, 3.9) |
| Combined PAF |  |  |  |  | 51.1 (31.4, 67.6) |
| **65-74** |  |  |  |  |  |
| Less education | 1.6 | 52.9 | 16.8 | 9.1 | 3.9 (2.4, 5.2) |
| Hearing loss | 1.9 | 16.6 | 54.2 | 32.8 | 14.1 (9.0, 17.3) |
| Traumatic brain injury | 1.8 | 38.1 | 13.0 | 9.4 | 4 (3.1, 4.9) |
| Hypertension | 1.6 | 42.6 | 53.2 | 24.2 | 10.4 (4.9, 14) |
| Excessive alcohol | 1.2 | 18.6 | 12.4 | 2.4 | 1.0 (0.6, 1.3) |
| Obesity | 1.6 | 64.9 | 32.9 | 16.5 | 7.1 (4.6, 8.2) |
| Smoking | 1.6 | 46.5 | 6.4 | 3.7 | 1.6 (0.6, 2.6) |
| Depression | 1.9 | 56.3 | 14.3 | 11.4 | 4.9 (4.0, 5.7) |
| Social isolation | 1.6 | 36.0 | 0.9 | 0.6 | 0.2 (0.1, 0.3) |
| Physical inactivity | 1.4 | 21.6 | 79.8 | 24.2 | 10.4 (7.0, 12.9) |
| Diabetes | 1.5 | 53.3 | 12.4 | 5.8 | 2.5 (2.4, 3.2) |
| Sleep disturbances | 1.2 | 45.4 | 39.7 | 7.4 | 3.2 (1.9, 3.8) |
| Combined PAF |  |  |  |  | 63.4 (40.7, 79.4) |
| **75-85** |  |  |  |  |  |
| Less education | 1.6 | 53.3 | 24.7 | 12.9 | 5.8 (3.8, 7.4) |
| Hearing loss | 1.9 | 10.7 | 81.2 | 42.2 | 19.0 (13.4, 21.6) |
| Traumatic brain injury | 1.8 | 24.4 | 8.9 | 6.7 | 3.0 (2.3, 3.6) |
| Hypertension | 1.6 | 42.0 | 59.8 | 26.4 | 11.9 (5.8, 15.5) |
| Excessive alcohol | 1.2 | 23.2 | 9.8 | 1.9 | 0.9 (0.5, 1.1) |
| Obesity | 1.6 | 48.4 | 26.0 | 13.5 | 6.1 (3.9, 7.0) |
| Smoking | 1.6 | 53.0 | 3.9 | 2.3 | 1.0 (0.4, 1.7) |
| Depression | 1.9 | 45.0 | 9.0 | 7.5 | 3.4 (2.8, 3.9) |
| Social isolation | 1.6 | 43.9 | 1.8 | 1.1 | 0.5 (0.3, 0.6) |
| Physical inactivity | 1.4 | 32.4 | 87.8 | 26.0 | 11.7 (8.2, 14.2) |
| Diabetes | 1.5 | 41.3 | 12.1 | 6.0 | 2.6 (2.5, 3.3) |
| Sleep disturbances | 1.2 | 31.0 | 41.7 | 7.7 | 3.5 (2.2, 4.1) |
| Combined PAF |  |  |  |  | 69.3 (46.2, 83.9) |

**Figure S6. Prevalence of modifiable risk factors for dementia in Canada by four age groups**

**Table S5. Sensitivity analysis for life-course model**

| Risk factors | RR | Communality (%) | Prevalence (%) | Unweighted PAF (%) | Weighted PAF (%) |
| --- | --- | --- | --- | --- | --- |
| Less education | 1.6 | 57.2 | 14.0 | 7.8 | 2.8 (1.7, 3.9) |
| Hearing loss | 1.9 | 45.6 | 21.0 | 15.9 | 5.8 (3.3, 8.3) |
| Traumatic brain injury | 1.8 | 33.9 | 15.0 | 10.7 | 3.9 (2.9, 4.8) |
| Hypertension | 1.6 | 63.6 | 30.0 | 15.3 | 5.6 (2.4, 8.3) |
| Excessive alcohol | 1.2 | 68.2 | 29.0 | 5.5 | 2.0 (1.2, 2.5) |
| Obesity | 1.6 | 63.3 | 31.0 | 15.7 | 5.7 (3.6, 6.8) |
| Smoking | 1.6 | 55.7 | 6.2 | 3.6 | 1.3 (0.5, 2.2) |
| Depression | 1.9 | 59.9 | 17.0 | 13.3 | 4.8 (3.9, 5.7) |
| Social isolation | 1.6 | 51.4 | 42.0 | 20.1 | 7.3 (4.7, 8.6) |
| Physical inactivity | 1.4 | 31.1 | 83.0 | 24.9 | 9.1 (6.0, 11.5) |
| Diabetes | 1.5 | 53.2 | 13.0 | 6.1 | 2.2 (2.1, 3) |
| Sleep disturbances | 1.2 | 50.1 | 40.0 | 7.4 | 2.7 (1.6, 3.4) |
| Combined PAF |  |  |  |  | 53.2 (33.9, 68.9) |

**Table S6. Sensitivity analysis by age groups**

| **Risk factors** | RR | Communality (%) | Prevalence (%) | Unweighted PAF (%) | Weighted PAF (%) |
| --- | --- | --- | --- | --- | --- |
| **Overall** |  |  |  |  |  |
| Less education | 1.6 | 54.1 | 12.5 | 7.0 | 2.4 (1.4, 3.3) |
| Hearing loss | 1.9 | 64.9 | 33.2 | 23.0 | 7.8 (4.6, 10.6) |
| Traumatic brain injury | 1.8 | 62.4 | 13.9 | 10.0 | 3.4 (2.5, 4.2) |
| Hypertension | 1.6 | 62.2 | 37.0 | 18.2 | 6.2 (2.7, 9.1) |
| Excessive alcohol | 1.2 | 48.9 | 27.0 | 5.1 | 1.7 (1.0, 2.2) |
| Obesity | 1.6 | 59.6 | 30.4 | 15.5 | 5.3 (3.3, 6.3) |
| Smoking | 1.6 | 60.2 | 10.7 | 6.0 | 2 (0.8, 3.3) |
| Depression | 1.9 | 44.0 | 17.4 | 13.5 | 4.6 (3.7, 5.4) |
| Social isolation | 1.6 | 30.5 | 21.3 | 11.4 | 3.9 (2.4, 4.7) |
| Physical inactivity | 1.4 | 55.3 | 76.6 | 23.4 | 8.0 (5.2, 10.3) |
| Type 2 diabetes | 1.5 | 51.9 | 8.5 | 4.1 | 1.4 (1.3, 1.9) |
| Sleep disturbances | 1.2 | 40.8 | 43.0 | 7.9 | 2.7 (1.6, 3.4) |
| Combined PAF |  |  |  |  | 49.4 (30.6, 64.7) |
| **45-54** |  |  |  |  |  |
| Less education | 1.6 | 49.7 | 7.2 | 4.2 | 1.8 (1.0, 2.5) |
| Hearing loss | 1.9 | 15.8 | 13.4 | 10.8 | 4.6 (2.5, 6.9) |
| Traumatic brain injury | 1.8 | 27.6 | 15.3 | 10.9 | 4.7 (3.5, 5.8) |
| Hypertension | 1.6 | 62.4 | 21.6 | 11.5 | 4.9 (2.0, 7.7) |
| Excessive alcohol | 1.2 | 51.1 | 30.6 | 5.8 | 2.5 (1.5, 3.1) |
| Obesity | 1.6 | 65.6 | 27.3 | 14.1 | 6.0 (3.7, 7.3) |
| Smoking | 1.6 | 54.3 | 13.7 | 7.6 | 3.3 (1.3, 5.3) |
| Depression | 1.9 | 51.7 | 17.4 | 13.6 | 5.8 (4.6, 6.9) |
| Social isolation | 1.6 | 30.9 | 6.0 | 3.5 | 1.5 (0.9, 1.9) |
| Physical inactivity | 1.4 | 33.2 | 71.5 | 22.2 | 9.5 (6.1, 12.4) |
| Type 2 diabetes | 1.5 | 62.0 | 4.3 | 2.1 | 0.9 (0.8, 1.2) |
| Sleep disturbances | 1.2 | 32.4 | 43.0 | 7.9 | 3.4 (2.0, 4.3) |
| Combined PAF |  |  |  |  | 49 (30.1, 65.3) |
| **55-64** |  |  |  |  |  |
| Less education | 1.6 | 59.6 | 12.4 | 6.9 | 2.6 (1.5, 3.5) |
| Hearing loss | 1.9 | 18.5 | 28.6 | 20.5 | 7.6 (4.4, 10.4) |
| Traumatic brain injury | 1.8 | 49.4 | 14.3 | 10.3 | 3.8 (2.9, 4.7) |
| Hypertension | 1.6 | 65.7 | 38.8 | 18.9 | 7.0 (3.1, 10.1) |
| Excessive alcohol | 1.2 | 65.6 | 28.3 | 5.4 | 2.0 (1.2, 2.5) |
| Obesity | 1.6 | 63.8 | 34.5 | 17.2 | 6.4 (4.1, 7.6) |
| Smoking | 1.6 | 68.5 | 11.7 | 6.6 | 2.5 (1.0, 3.9) |
| Depression | 1.9 | 62.7 | 18.3 | 14.2 | 5.3 (4.3, 6.1) |
| Social isolation | 1.6 | 32.5 | 20.9 | 11.1 | 4.1 (2.5, 5.0) |
| Physical inactivity | 1.4 | 31.8 | 76.8 | 23.5 | 8.8 (5.8, 11.2) |
| Type 2 diabetes | 1.5 | 61.3 | 10.3 | 4.9 | 1.8 (1.7, 2.4) |
| Sleep disturbances | 1.2 | 55.5 | 45.4 | 8.3 | 3.1 (1.9, 3.8) |
| Combined PAF |  |  |  |  | 55 (34.4, 71.3) |
| **65-74** |  |  |  |  |  |
| Less education | 1.6 | 49.2 | 16.6 | 9.1 | 3.4 (2.1, 4.5) |
| Hearing loss | 1.9 | 27.5 | 54.1 | 32.7 | 12.4 (8.0, 15.2) |
| Traumatic brain injury | 1.8 | 54.4 | 13.0 | 9.4 | 3.6 (2.7, 4.3) |
| Hypertension | 1.6 | 52.5 | 53.2 | 24.2 | 9.2 (4.3, 12.4) |
| Excessive alcohol | 1.2 | 46.2 | 23.1 | 4.4 | 1.7 (1.0, 2.1) |
| Obesity | 1.6 | 62.2 | 32.9 | 16.5 | 6.3 (4.0, 7.3) |
| Smoking | 1.6 | 66.6 | 6.4 | 3.7 | 1.4 (0.6, 2.3) |
| Depression | 1.9 | 69.2 | 14.9 | 11.8 | 4.5 (3.7, 5.2) |
| Social isolation | 1.6 | 34.9 | 37.2 | 18.3 | 6.9 (4.5, 8.0) |
| Physical inactivity | 1.4 | 23.0 | 80.0 | 24.2 | 9.2 (6.2, 11.4) |
| Type 2 diabetes | 1.5 | 55.5 | 12.4 | 5.8 | 2.2 (2.1, 2.9) |
| Sleep disturbances | 1.2 | 65.8 | 39.8 | 7.4 | 2.8 (1.7, 3.4) |
| Combined PAF |  |  |  |  | 63.5 (41.0, 78.8) |
| **75-85** |  |  |  |  |  |
| Less education | 1.6 | 44.4 | 24.7 | 12.9 | 5.0 (3.3, 6.3) |
| Hearing loss | 1.9 | 37.1 | 81.2 | 42.2 | 16.3 (11.6, 18.5) |
| Traumatic brain injury | 1.8 | 20.4 | 8.9 | 6.7 | 2.6 (2.0, 3.1) |
| Hypertension | 1.6 | 32.8 | 59.8 | 26.4 | 10.2 (5.1, 13.3) |
| Excessive alcohol | 1.2 | 13.6 | 17.5 | 3.4 | 1.3 (0.8, 1.6) |
| Obesity | 1.6 | 47.2 | 25.9 | 13.5 | 5.2 (3.4, 6.0) |
| Smoking | 1.6 | 54.5 | 3.9 | 2.3 | 0.9 (0.4, 1.4) |
| Depression | 1.9 | 57.2 | 18.8 | 14.4 | 5.6 (4.8, 6.2) |
| Social isolation | 1.6 | 27.3 | 50.5 | 23.2 | 9.0 (6.2, 9.9) |
| Physical inactivity | 1.4 | 38.7 | 87.8 | 26.0 | 10.1 (7.1, 12.1) |
| Diabetes | 1.5 | 37.6 | 12.1 | 5.7 | 2.2 (2.2, 2.8) |
| Sleep disturbances | 1.2 | 42.3 | 41.6 | 7.7 | 3.0 (1.9, 3.5) |
| Combined PAF |  |  |  |  | 71.4 (48.7, 84.8) |

**Table S7. Sensitivity analysis by sex**

|  | Female |  |  |  |  | Male |  | |  | |  |
| --- | --- | --- | --- | --- | --- | --- | --- | --- | --- | --- | --- |
| Risk factors | Communality (%) | Prevalence (%) | Unweighted PAF (%) | Weighted PAF (%) |  | Communality (%) | Prevalence (%) | Unweighted PAF (%) | | Weighted PAF (%) | |
| Less education | 51.2 | 12.0 | 6.7 | 2.6 (1.6, 3.6) |  | 57.2 | 13.1 | 7.3 | | 2.6 (1.6, 3.6) | |
| Hearing loss | 54.3 | 29.6 | 21.1 | 8.2 (4.8, 11.1) |  | 45.6 | 37.1 | 25.0 | | 9.1 (5.5, 12) | |
| Traumatic brain injury | 16.8 | 10.3 | 7.6 | 3.0 (2.2, 3.6) |  | 33.9 | 17.8 | 12.5 | | 4.5 (3.5, 5.5) | |
| Hypertension | 52.5 | 35.4 | 17.5 | 6.8 (3.0, 9.9) |  | 63.6 | 38.8 | 18.9 | | 6.9 (3.1, 9.9) | |
| Excessive alcohol | 24.2 | 19.9 | 3.8 | 1.5 (0.9, 1.9) |  | 68.2 | 34.9 | 6.5 | | 2.4 (1.4, 3.0) | |
| Obesity | 59.2 | 30.2 | 15.3 | 6.0 (3.8, 7.1) |  | 63.3 | 30.7 | 15.6 | | 5.7 (3.6, 6.7) | |
| Smoking | 57.4 | 10.6 | 6.0 | 2.3 (1.0, 3.7) |  | 55.7 | 10.7 | 6.1 | | 2.2 (0.9, 3.6) | |
| Depression | 52.7 | 20.6 | 15.6 | 6.1 (5.0, 7.0) |  | 59.9 | 13.9 | 11.1 | | 4.0 (3.3, 4.8) | |
| Social isolation | 46.4 | 26.0 | 13.5 | 5.3 (3.3, 6.3) |  | 51.4 | 16.2 | 8.9 | | 3.2 (2.0, 4.0) | |
| Physical inactivity | 25.6 | 80.0 | 24.2 | 9.5 (6.3, 11.9) |  | 31.1 | 72.8 | 22.6 | | 8.2 (5.4, 10.5) | |
| Diabetes | 56.6 | 7.2 | 3.5 | 1.4 (1.3, 1.8) |  | 53.2 | 10.0 | 4.7 | | 1.7 (1.6, 2.3) | |
| Sleep disturbances | 48.4 | 42.4 | 7.8 | 3.0 (1.9, 3.7) |  | 50.1 | 43.8 | 8.1 | | 2.9 (1.8, 3.6) | |
| Combined PAF | |  |  | 55.7 (35.2, 71.6) |  |  |  |  | | 53.4 (33.5, 69.4) | |

**References**

1. Raina P, Wolfson C, Kirkland S, et al. Cohort Profile: The Canadian Longitudinal Study on Aging (CLSA). *Int J Epidemiol*. Dec 1 2019;48(6):1752-1753j. doi:10.1093/ije/dyz173
